# RNA-Seq-Mediated Transcriptome Analysis of a Fiberless Mutant Cotton and Its Possible Origin Based on SNP Markers

**DOI:** 10.1371/journal.pone.0151994

**Published:** 2016-03-18

**Authors:** Qifeng Ma, Man Wu, Wenfeng Pei, Xiaoyan Wang, Honghong Zhai, Wenkui Wang, Xingli Li, Jinfa Zhang, Jiwen Yu, Shuxun Yu

**Affiliations:** 1 State Key Laboratory of Cotton Biology, Institute of Cotton Research of the Chinese Academy of Agricultural Sciences, Anyang, China; 2 College of Biology and Food Technology, Anyang Institute of Technology, Anyang, China; 3 Department of Plant and Environmental Sciences, New Mexico State University, Las Cruces, New Mexico, United States of America; Texas Tech University, UNITED STATES

## Abstract

As the longest known single-celled trichomes, cotton (*Gossypium* L.) fibers constitute a classic model system to investigate cell initiation and elongation. In this study, we used a high-throughput transcriptome sequencing technology to identify fiber-initiation-related single nucleotide polymorphism (SNP) markers and differentially expressed genes (DEGs) between the wild-type (WT) Upland cotton (*G*. *hirsutum*) Xuzhou 142 and its natural fuzzless-lintless mutant Xuzhou 142 *fl*. Approximately 700 million high-quality cDNA reads representing over 58 Gb of sequences were obtained, resulting in the identification of 28,610 SNPs—of which 17,479 were novel—from 13,960 expressed genes. Of these SNPs, 50% of SNPs in *fl* were identical to those of *G*. *barbadense*, which suggests the likely origin of the *fl* mutant from an interspecific hybridization between Xuzhou 142 and an unknown *G*. *barbadense* genotype. Of all detected SNPs, 15,555, 12,750, and 305 were classified as non-synonymous, synonymous, and pre-terminated ones, respectively. Moreover, 1,352 insertion/deletion polymorphisms (InDels) were also detected. A total of 865 DEGs were identified between the WT and *fl* in ovules at −3 and 0 days post-anthesis, with 302 candidate SNPs selected from these DEGs for validation by a high-resolution melting analysis and Sanger sequencing in seven cotton genotypes. The number of genotypic pairwise polymorphisms varied from 43 to 302, indicating that the identified SNPs are reliable. These SNPs should serve as good resources for breeding and genetic studies in cotton.

## Introduction

Upland cotton, *Gossypium hirsutum* L., is native to Mexico, the West Indies, and northern South America. This species, which is grown in more than 80 countries, supplies almost 95% of the natural fiber, the raw material for different textiles [1]. Cotton fibers, which originate from the outer epidermal layer of the seeds, are classified into lint and fuzz types. Lint fiber formation begins at anthesis, i.e., 0 to 2 days post-anthesis (DPA). By contrast, the fuzz fibers (called linters) initiate growth after 3 DPA and cannot be separated from the seeds by ginning [2]. Because the number of lint fiber initials affects cotton lint yield, much research effort has been directed at revealing the regulatory mechanisms of fiber initiation at the individual gene, transcriptome, and proteome levels [3–5]. In addition, genome-wide association studies and quantitative trait locus analyses based on linkage maps of molecular markers have been conducted to facilitate marker-assisted selection for fiber traits [6–8]. Cotton yield and fiber quality are complex quantitative traits, however, and the number of candidate gene-based molecular markers for key fiber traits is currently limited.

Genetic mapping of major fiber mutants in cotton is an important approach for isolation of fiber-related genes and to provide clues to their functions. An Upland cotton fuzzless-lintless Xuzhou 142 *fl* mutant, which possesses no fiber (i.e., no lint and fuzz), was originally discovered in its wild type (WT), the cultivar Xuzhou 142 with lint and fuzz fibers [9]. The two lines are thus thought to be near-isogenic lines, although there is no molecular confirmation. The *fl* mutant phenotype is controlled by two pairs of recessive genes (*li3li3n2n2*), but the location of the *fl* gene locus is still unknown [10]. Although *fl* arose as a natural isolate from Xuzhou 142, its morphological characteristics have cast doubt on the isogenic nature of the two genotypes. Although a detailed genetic comparison of the two genotypes is needed, such an analysis is hindered by the nature of the complex cotton genome. Because the tetraploid genome of cotton is relatively large—approximately 2.5 Gb [11] and the level of intraspecific DNA polymorphism is low, the development of polymorphic molecular markers for this purpose is a challenging task [12].

To date, many types of molecular markers, such as amplified fragment length polymorphisms, random amplified polymorphic DNAs, restriction fragment length polymorphisms, restriction-site-associated DNA markers, simple sequence repeats, and single nucleotide polymorphisms (SNPs), have been developed for applications to cotton research [13]. To construct a saturated genetic map in complex genomes such as cotton, however, a large number of molecular markers are required. Because of their advantages over other marker systems, including higher genomic density, genotyping efficiency, and reproducibility, SNP markers are the current choice for population mapping [14]. Many studies related to the diversity, characterization, and mapping of SNPs in the *Gossypium* genome have been conducted. For example, An et al. identified gene family-targeted SNP markers [15], while Lu et al. reported the development of fiber gene SNP markers based on simple-strand conformation polymorphisms [16]. Deynze et al. developed 270 single-copy polymorphic loci in cotton based on expressed sequence tags [17]. Byers et al. found 11,834 SNPs between a commercial *G*. *hirsutum* cultivar, Acala Maxxa, and the wild landrace TX2094, which encompass the breadth of *G*. *hirsutum* diversity [18]. In addition, an international collaborative effort has recently focused on the development of a 63K SNP chip based on the Illumina Infinium genotyping assay for the mapping of *G*. *hirsutum* and inter-specific populations of *G*. *hirsutum* × *G*. *barbadense* [19]. To date, 183,035 SNPs have been deposited in the CottonGen database, the largest repository of cotton SNP markers and mapping information [20]. These existing efforts represent important early advances in cotton genomics and SNP discovery. Nevertheless, the development of additional SNP identification and typing methodologies is needed for effective SNP discovery among elite cultivars with relatively narrow pedigrees and to provide more genuine varietal SNPs for marker-assisted breeding within *G*. *hirsutum*. Insertion/deletions (InDels) cause similar levels of variation as SNPs, but with great diversity in size. Moreover, InDels can be distinguished easily and also become popular as genetic markers for mapping and other genetic research in crops.

The application of next-generation sequencing, including Roche 454 and Illumina Genome Analyzer systems, can accelerate the identification of new markers and genes and has a great potential to determine the intricacies of cotton fiber differentiation and initiation. Even though the 454 pyrosequencing technology has been applied to cotton genomics [21], the Illumina sequencing platform has gained a wider acceptance because of its lower cost and higher sequencing quality and throughput [22]. In spite of the potential of next-generation sequencing, SNP discovery using this technique has not been reported for Xuzhou 142 and its *fl* mutant. In this study, we therefore applied next-generation RNA sequencing (RNA-seq) for the development of fiber initiation-related SNP markers based on an analysis of differentially expressed genes (DEGs) between the WT and the *fl* mutant. The gene markers identified in this study represent a valuable resource for genetic diversity assessment and mapping of agronomic traits leading to the isolation of the lintless and fuzzless genes in the *fl* mutant.

## Materials and Methods

### Plant materials

Upland cotton *G*. *hirsutum* ‘Xuzhou 142’ and its *fl* mutant line were grown at the Institute of Cotton Research, Chinese Academy of Agricultural Sciences, Anyang, Henan Province, China. Three replications of the two genotypes were arranged side by side, with 10 plants per replication per genotype. Ovules from flower buds at −3 DPA and flowers at 0 DPA were collected, immediately dissected, frozen in liquid nitrogen, and stored at −80°C until use. Along with Xuzhou 142 and *fl*, five additional Upland cotton genotypes were used for SNP validation: Upland genetic standard TM-1, backcross inbred line (BIL)-7, BIL-13, Zhong-58, and Zhong-58 mutant. The BIL-13 with longer fiber length and BIL-7 were derived from Giza 75 (G. *barbadense*) and SG 747 (G. *hirsutum*) through two generations of backcrossing (BC2) with SG 747 as the recurrent parent, followed by self-pollination for four generations. Zhong-58 was a commercial cultivar while Zhong-58 virescent mutant was derived from cotton cultivar Zhong-58, showing yellow leaves at the seeding stage and longer fibers than its wild type.

### RNA isolation and transcriptome sequencing

Total RNA was isolated using an RNAprep Pure Plant kit (Tiangen, Beijing, China) according to the manufacturer’s instructions. After digestion of residual DNA with DNase I (New England Biolabs, Ipswich, MA, USA), RNA quality and quantity were measured on a Nanodrop 2000 instrument (Thermo Fisher Scientific, Wilmington, DE, USA) and an Agilent 2100 Bioanalyzer (Agilent Technologies, Palo Alto, CA, USA). Samples with 260/280 ratios of 1.9 to 2.1 and RNA integrity numbers ≥ 8 were used in subsequent analyses.

Poly(A) mRNA was isolated using oligo(dT) beads and then sheared into short fragments using a fragmentation buffer [23]. The short fragments were then used as templates to synthesize cDNA using random hexamer primers and reverse transcriptase. Twelve paired-end cDNA libraries were constructed from RNAs of cotton ovules with insert sizes ranging from 200 to 700 bp. The libraries were then sequenced based on a 90-bp read length on an Illumina Genome Analyzer (HiSeqTM2000 Sequencing System).

The RNA-seq data used for this analysis have been submitted to the National Center for Biotechnology Information-Sequence Read Archive database (SRA) and assigned the identifier SRP056184.

### Sequence alignment and SNP/InDel calling

After adaptor trimming and removal of low-quality reads, RNA-seq reads were stringently aligned against the Upland cotton (AADD) TM-1 genome using the Burrows-Wheeler Alignment tool [11, 24]. SNPs/InDels were called using the Genome Analysis Toolkit [25] based on three criteria: read depth ≥ 30, mapping quality ≥ 20, non-ambiguous reference base and the length of InDel ≥ 2.

### Identification of DEGs

Gene expression levels were measured by RNA-seq quantification analysis using the Noiseq package [26]. The following criteria were used to identify DEGs: false discovery rate ≤ 0.001, |log2 ratio| ≥ 1 and probability ≥ 0.85.

The Blast2GO program, with a cutoff value of 1 × 10^−5^, was used to obtain DEG Gene Ontology (GO) annotations [27]. This analysis mapped all DEGs to GO terms in the database and counted the number of DEGs associated with each term. The WEGO program was then applied to plot GO functional classifications of the DEGs [28].

### SNP validation

To validate SNP markers, genomic DNA was extracted from the WT, *fl*, and the other five Upland cotton genotypes as described by Zhang and Stewart [29]. Using primers designed from DEGs having SNPs between the WT and *fl*, we amplified 302 SNPs to generate 200–500-bp fragments (S1 Table). The PCR products from the seven cotton genotypes were combined and sequenced on a Miseq platform (Illumina, San Diego, CA, USA). A set of SNPs was also analyzed using high-resolution melting (HRM) and Sanger sequencing. The 20-μL HRM reaction mixture consisted of the following regents: 10.0 μL of 2× LightCycler 480 HRM master mix, 0.4 μL of each 10 μM primer, 2 μL MgCl_2_ (25 mM), 2 μL DNA (25 ng μL^−1^), and 5.2 μL deionized water. A touchdown PCR was initiated with melting for 10 min at 95°C, followed by 42 cycles of 95°C for 10 s, a touchdown cycling step of annealing from 65 to 56°C for 15 s, and 72°C for 20 s. After amplification, the PCR products were heated from 65 to 95°C and data was acquired at a rate of 25 scans per °C. To further validate the identified SNPs by Sanger sequencing, five SNPs were randomly selected from those successfully genotyped by HRM. Their associated PCR products were cloned into a pMD18-T vector (Takara, Dalian, China) and transformed into *Escherichia coli* (DH5a). Subsequently, 6–10 clones were sequenced using the Sanger sequencing method.

## Results

### SNP identification

**Fig 1:**
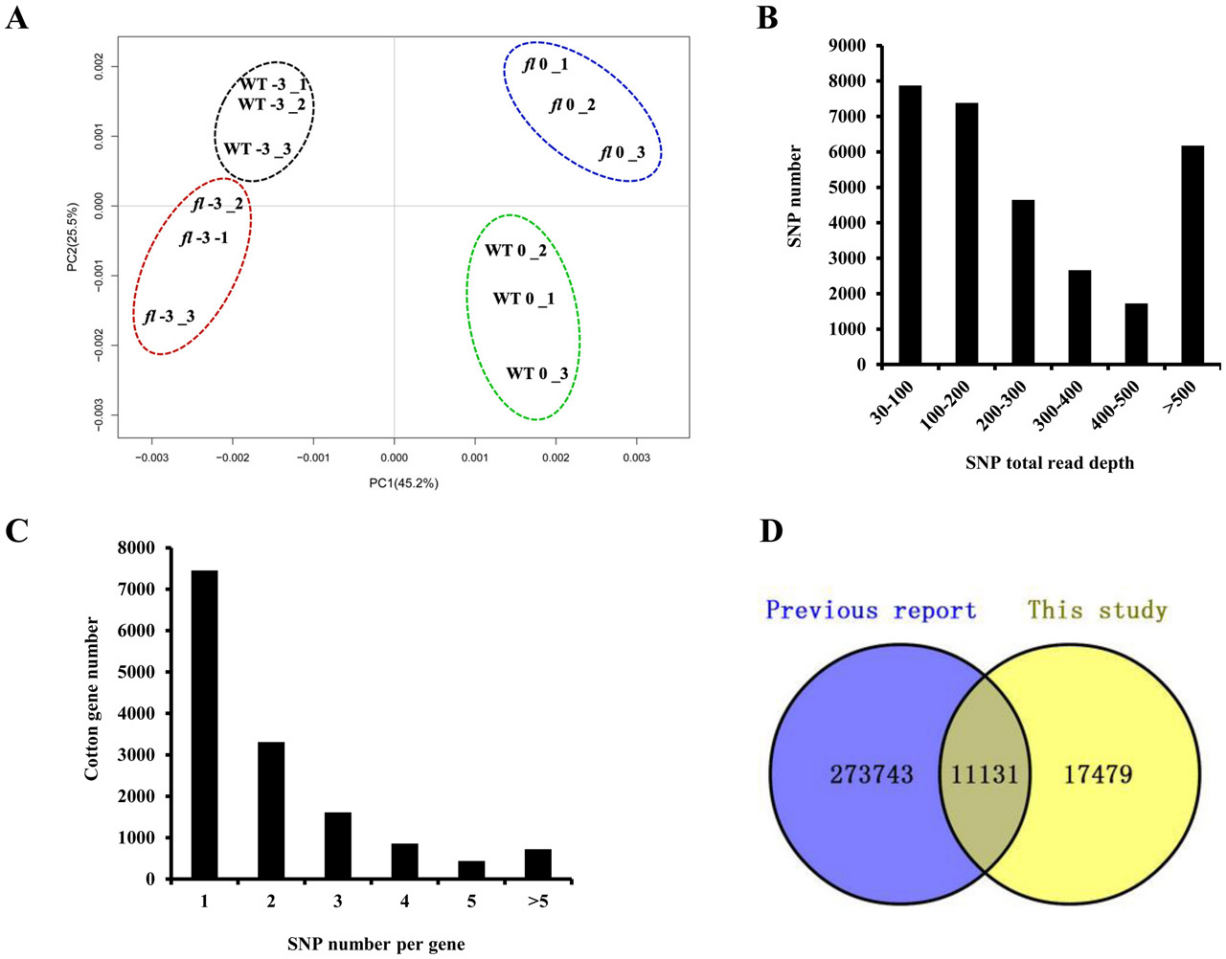
Overview of single nucleotide polymorphism (SNP) data generated in this study. (A) Principal component analysis of 12 sample datasets. (B) Frequency distribution of SNP total read depth. (C) Distribution of SNPs per gene. (D) Venn diagram comparison of SNPs identified in this study vs. SNPs previously reported in cotton.

To reduce the dimensionality of the generated dataset, principal component analysis was used to summarize the gene expression data features of the 12 transcriptomes, i.e., the three biological replicates of −3 and 0 DPA ovules of the WT and *fl* (Fig 1A). After filtering out low-quality sequences, a total of 699,827,334 90-bp clean reads consisting of 60 Gbp and corresponding to 635-fold coverage of the expressed genome of upland cotton were obtained from the 12 libraries. The majority of the reads (≥ 76%) were mapped to the TM-1 reference genome (Table 1). As a result, a total of 56,765 genes were identified, indicating that 73.8% (76,943) of predicted genes in the Upland cotton genome were expressed in the ovules.

**Table 1:** Statistical summary of clean reads from RNA sequencing of 12 sample libraries.

Sample name	Clean reads	Mapped reads	Genome map rate
*fl* 0_1	58,658,090	45,348,569	77.31%
*fl* 0_2	58,190,110	45,877,082	78.84%
*fl* 0_3	58,522,172	46,396,377	79.28%
WT 0_1	58,349,536	44,678,239	76.57%
WT 0_2	58,368,912	44,663,891	76.52%
WT 0_3	58,274,832	45,401,921	77.91%
*fl* -3_1	58,259,328	45,191,760	77.57%
*fl* -3_2	58,223,122	44,779,403	76.91%
*fl* -3_3	58,243,860	45,156,464	77.53%
WT -3_1	58,231,532	45,269,192	77.74%
WT -3_2	58,304,140	45,325,638	77.74%
WT -3_3	58,201,700	45,583,571	78.32%

The average sequencing depth of the WT and *fl* was 624 and 646, respectively, indicating a sufficient sequencing depth for SNP detection. Of the 28,610 SNPs detected in 13,960 genes, 12,750 were synonymous and 15,860 were non-synonymous (including 305 nonsense SNPs) (S2 Table). Transition SNPs, including 31.6% A/G and 32.9% C/T, were the most common type, while transversion SNPs were represented by 8.8% A/C, 9.9% A/T, 8.1% G/C, and 8.7% G/T mutations (Table 2). The proportion of transitions to transversions was approximately 1.8. Moreover, 660 insertions and 692 deletions were detected, ranging from 2 to 21 bp in length. Insertions and deletions with 2 bp in length were predominant (402 and 394 cases, respectively), which were included in SNPs in many studies. Longer insertions or deletions were rare (S2 Table).

**Table 2:** Classification of identified single nucleotide polymorphisms.

SNP type	Number	Percentage
Transition		
A/G	9,003	31.60%
C/T	9,463	32.90%
Transversion		
A/C	2,520	8.80%
A/T	2,847	9.90%
G/C	2,273	8.10%
G/T	2,504	8.70%

A statistical summary of SNP read depths is given in Fig 1B. The average SNP read depth was 473. SNPs with read depths ranging from 30 to 300 accounted for 65.9% of detected SNPS, with read depths between 300 and 500 represented by 14.2%. The detected SNPs were distributed in 13,960 genes, the majority (99.5%) of which contained less than 10 SNPs (Fig 1C). An average of two SNPs per gene was identified. We also compared the SNPs detected in our study with previously reported novel SNPs. Among our detected SNPs, 17,479 SNPs were not found in SNP public databases (i.e., CottonGen and NCBI) (Fig 1D), implying that these SNPs are novel and some may be related to fiber initiation. A total of 25,790 of the 28,610 SNPs could be found in the *G*. *barbadense* genome, and 58.8% of these (15,762) showed polymorphisms from Upland cotton. Moreover, 50% of the SNPs from *fl* were the same as those in *G*. *barbadense*. These results clearly suggest that *fl* may have originated from an interspecific cross between Xuzhou 142 and an unknown *G*. *barbadense* genotype.

### Analysis of DEGs with SNPs

**Fig 2:**
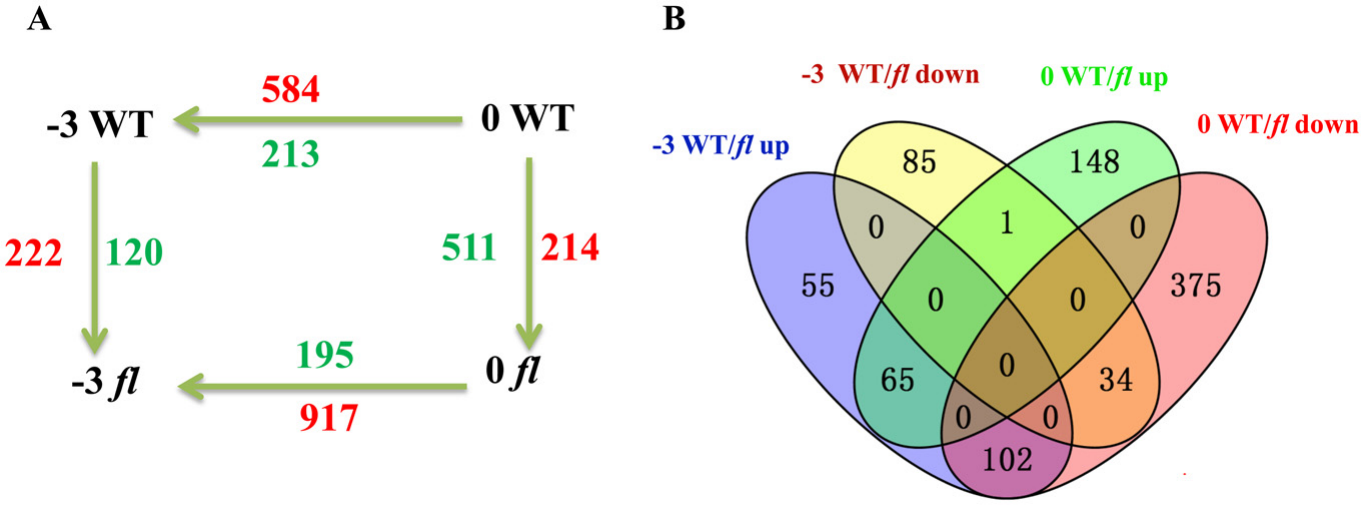
Summary of identified differentially expressed genes (DEGs). (A) Number of DEGs during different stages of ovule development and between wild-type (WT) upland cotton cultivar Xuzhou 142 and its *fl* mutant. The number of up- and down-regulated genes are indicated in red and blue, respectively. For example, 222 genes were up-regulated and 120 were down-regulated 3 days before anthesis (−3 DPA) in the WT compared with *fl*. (B) Venn diagram representation of DEGs between the WT and *fl*.

A total of 865 genes differentially regulated between the WT and *fl* were identified by comparing sequence reads between −3 DPA WT and −3 DPA *fl* and between 0 DPA WT and 0 DPA *fl* (S3 Table). Compared with *fl*, 222 genes were up-regulated and 120 were down-regulated in the WT at −3 DPA, while 214 were up-regulated and 511 were down-regulated in the WT at 0 DPA (Fig 2A). Among the 865 DEGs, the abundances of 65 were significantly up-regulated and 34 were down-regulated at both −3 and 0 DPA in the WT (Fig 2B). Interestingly, the expression dynamics of transcription factor genes during fiber initiation were particularly evident in our data, as 106 transcription factor DEGs were detected at the fiber initiation stage. The DEGs identified in this study also included certain important fiber-initiation-related genes, such as genes encoding bHLH, R2R3 MYB, auxin-responsive protein, calcium ion binding protein, and serine/threonine-protein kinase. The expression patterns of some DEGs were consistent with those uncovered by quantitative real-time PCR in our studies.

**Fig 3:**
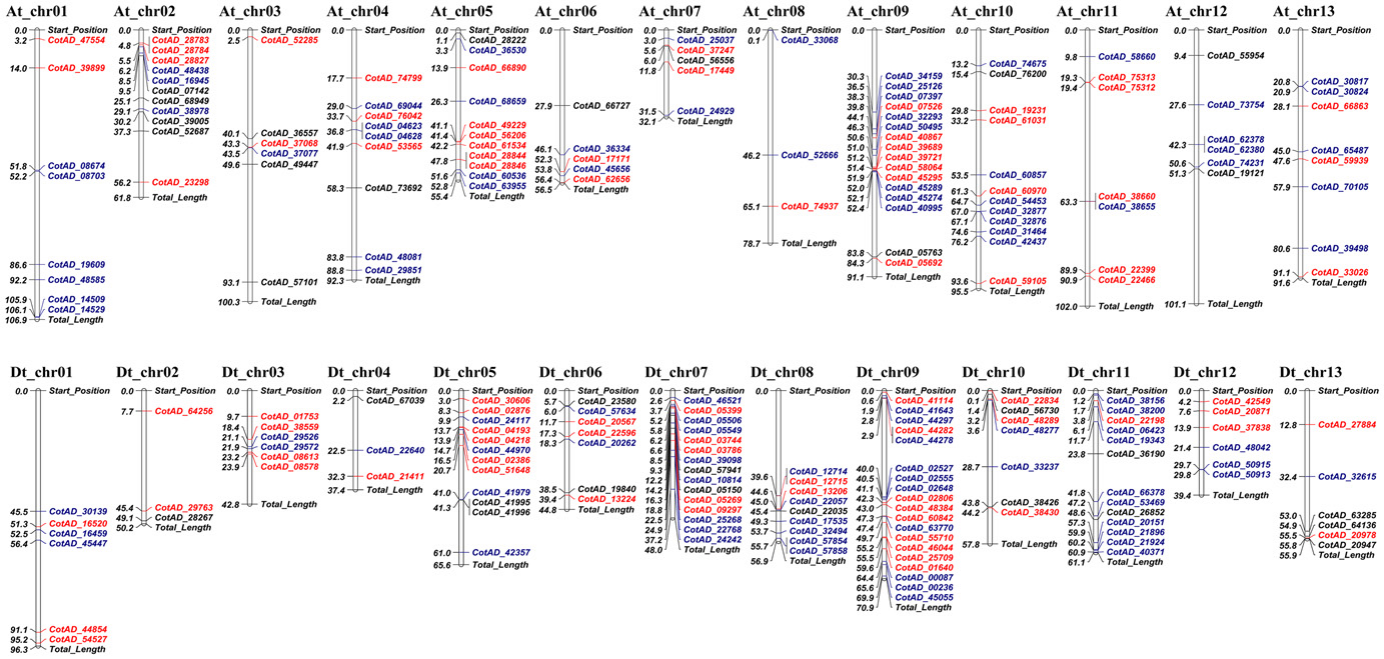
Physical map of single nucleotide polymorphisms (SNPs) in differentially expressed genes (DEGs). Genes up- or down-regulated in wild-type (WT) *Gossypium hirsutum* ‘Xuzhou 142’ relative to the *fl* mutant are indicated in red and blue, respectively. Genes differentially expressed between −3 and 0 days post-anthesis in the WT are shown in black.

Because most of the SNPs/InDels identified between Xuzhou 142 and its *fl* mutant are not genetically related to the fiberlss trait due to the interspecific origin of *fl*, only these SNPs/InDels within DEGS were focused. A total of 219 SNP/InDel-containing DEGs were mapped to the 26 chromosomes of tetraploid cotton. The number of DEGs with SNPs ranged from 3 to 17 per chromosome (Fig 3). In a previous study using aneuploids, the *n2* locus was assigned to chromosome 26, whereas Rong et al. reported that *n2* was linked to several markers on its homoeolog, i.e., chromosome 12. In our study, 12 genes could be mapped to both of these chromosomes. This finding may lead to accelerated genetic dissection of cotton fiber development.

### SNP classification and annotation

**Fig 4:**
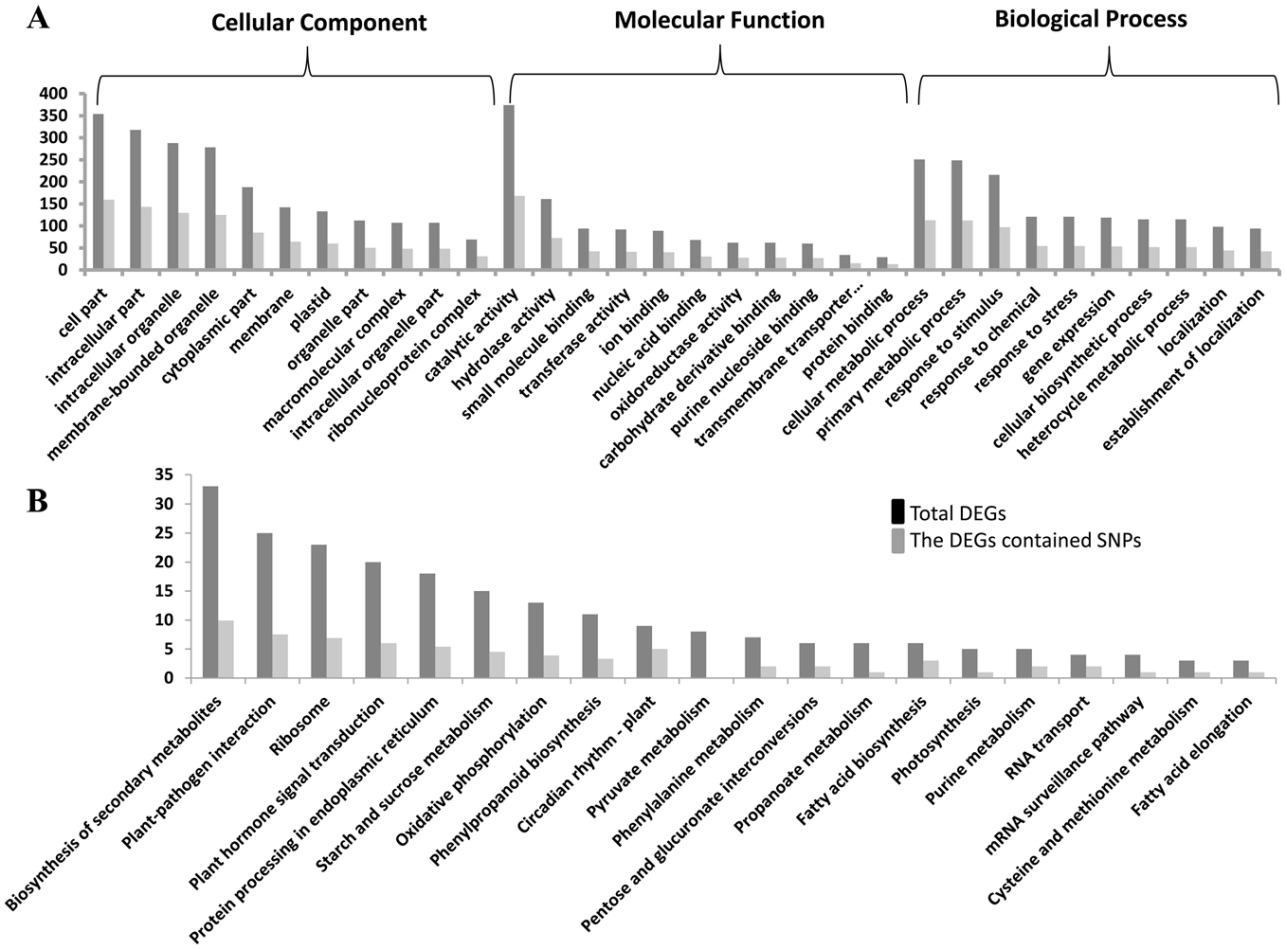
Gene Ontology (A) and Kyoto Encyclopedia of Genes and Genomes pathway (B) classifications of differentially expressed genes containing single nucleotide polymorphisms.

Blast2GO was used to classify the DEGs into molecular function, biological process, and cellular component plant GO categories. DEGs that showed significant homology to genes in the NCBI non-redundant database were selected for GO annotation. Among the DEGs with SNPs, information related to biological process, cellular component, and molecular function was obtained for 113, 159, and 168 genes, respectively (Fig 4A). In this study, most of the identified DEGs were involved in binding and transferase activity, consistent with the findings of our previous investigation.

To identify metabolic pathways active during fiber initiation, we mapped the DEGs to Kyoto Encyclopedia of Genes and Genomes reference pathways. As a result, 442 DEGs were mapped to 108 pathways. Pathways related to biosynthesis of secondary metabolites were the most abundant, followed by plant-pathogen interaction, ribosomes, and plant hormone signal transduction (Fig 4B).

### SNP validation

**Fig 5:**
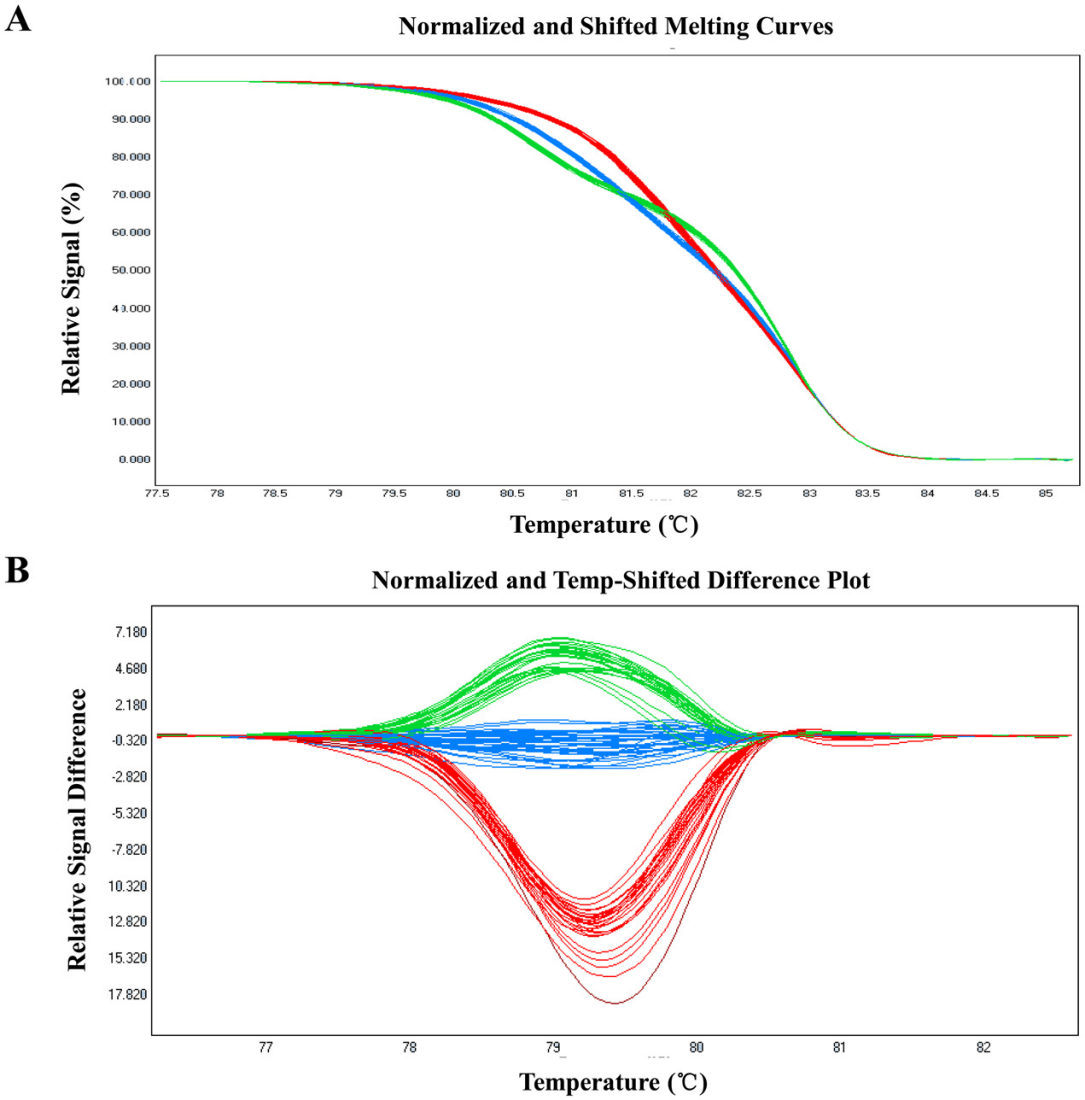
High-resolution melting analysis to confirm the presence of single nucleotide polymorphisms. (A) Original melting curves. (B) Melting curves after logarithm calculations. Blue, green, and red curves correspond to wild-type, *fl* mutant, and heterozygous genotypes, respectively.

The 302 validated SNP markers derived from the DEGs were also detected in five other cotton genotypes (Table 3). The highest levels of polymorphism were found between TM-1 and BIL13 compared with 222; the lowest level, 43 polymorphic SNP markers, was observed between Zhong-58 and its virescent mutant. A number of SNPs could be found simultaneously among the seven genotypes, indicating that these polymorphic markers are reliable. HRM analysis, which generates obvious patterns between heterozygous and homozygous sites based on different melting curve shapes, was also used for validation of the SNPs. All samples with different genotypes were automatically grouped with the LightCycler 480 Gene Scanning software, which successfully genotyped all SNPs between the WT and *fl*. An example of an HRM assay difference plot in which homozygous genotypes of the heat shock protein (CotAD_12715) are separated from heterozygous ones in an F_2_ segregating population is shown in Fig 5. In addition, the PCR amplicons of ten randomly selected SNP-containing candidate gene fragments were sequenced using the Sanger method, which confirmed that all amplicons contained the predicted variation.

**Table 3:** Validation of 302 single nucleotide polymorphisms among seven cotton cultivars.

	TM-1	WT	*fl*	BIL7	BIL13	Zhong-58	Zhong-58 mutant
TM-1		172	130	220	222	136	115
WT	56.95%		302	120	126	152	147
*fl*	43.05%	100.00%		182	176	150	155
BIL7	72.85%	39.74%	60.26%		94	176	191
BIL13	73.51%	41.72%	58.28%	31.13%		168	179
Zhong-58	45.03%	50.33%	49.67%	58.28%	55.63%		43
Zhong-58 mutant	38.08%	48.68%	51.32%	63.25%	59.27%	14.24%	

## Discussion

Because of their advantages of genome-wide coverage, data quality, high genotyping efficiency and analytical simplicity [30], SNP markers have rapidly become the accepted choice for many applications in genomic and genetic studies. One aim of SNP discovery applied to genome-wide transcriptome data is the acquisition of SNPs directly associated with traits of interest, such as growth parameters or disease resistance [31, 32]. The goal of the present research was to discover a large number of SNPs between two cotton genotypes through transcriptome sequencing. Another goal was the identification of fiber-initiation-related SNPs to facilitate understanding of the mechanism of cotton fiber initiation. To generate many SNPs, 12 sets of transcriptomic data were generated. Genome coverage and read depth are key parameters affecting SNP prediction accuracy. In this study, the average read depth of SNP positions was 473, which was adequate to guarantee the accuracy of the discovered SNPs. Among the putative SNPs, 26,744 (93.5%) were identified from genes with coverage of more than 50 reads; this suggests that most identified SNPs were covered at a sufficient read depth and probably represent ‘true’ SNPs. A further analysis revealed that 17,479 SNPs were detected for the first time, and our study thus supply candidate gene SNPs for marker development that will be useful for further studies in genetics, genomics, evolution, and molecular ecology in cotton. Most importantly, a larger than expected number of SNPs was detected between *fl* and its isogenic WT, suggesting the non-isogenic nature of the two genotypes. Therefore, it should be recognized that most of the differentially expressed genes (DEGs) identified from our study were not related to the fuzzless and lintless genes and their expressions. *Gossypium barbadense*, also known as long-staple cotton, generally has no fuzz (i.e., fuzzless), a trait controlled by recessive *n2n2*. A comparative analysis revealed that more than half of the SNPs in *fl* were the same as those in *G*. *barbadense*, indicating that most of the SNPs identified in this study involved polymorphisms between Upland cotton and *G*. *barbadense* genomes. Consequently, the *fl* mutant may have arisen from interspecific hybridization between Xuzhou 142 and a *G*. *barbadense* genotype that contributed the recessive *n2n2* gene to *fl*. Xuzhou 142 may carry another recessive gene *li3li3* and its combination with *n2n2* gave rise to the fuzzless and lintless mutant.

The top 20 genes with the highest number of SNPs are listed in S2 Table. For instance, the NB-ARC domain-containing disease resistance (R) protein (CotAD_22386) and homeodomain protein HOX3 (CotAD_48384) have relatively large numbers of SNPs per gene, 29 and 27, respectively. The NB-ARC domain-containing gene, which was up-regulated in the WT at −3 DPA, is responsible for the autoactivation and multimerization of R proteins during defense responses [33]. This gene exhibits higher ratios of nonsynonymous-to-synonymous SNPs than other genes. Although first discovered in cotton fiber mutants, this phenomenon is actually very common and can be found in other crops such as sorghum [34], rice [35], and soybean [36]. The high nonsynonymous-to-synonymous SNP ratio in this gene suggests a dynamic evolution to combat pathogens and is also an important component of plant defenses [37]. The gene with the second highest SNP number, *ChHOX3*, was found to control cotton fiber elongation in a previous survey of 281 cotton cultivars [38]. In that study, two SNPs were detected at positions 2,560 (C/T) and 2,761 (G/A). A further association analysis indicated that the two SNPs were significantly correlated with fiber length and uniformity. GhHOX3 interacts with a homeodomain leucine zipper protein (GhHD1) and binds to the DELLA protein GhSLR1, resulting in reduced production of gibberellin. Moreover, *GhHOX3* overexpression leads to longer fibers, whereas its silencing significantly reduces fiber length (> 80%). Those results indicate that SNP variation in *GhHOX3* influences gibberellin signaling and thus affects fiber cell elongation.

Transcription factors, which often form multi-protein complexes, bind with specific DNA sequences to increase or decrease gene transcription [39]. To understand the regulatory networks involved in various signaling and metabolic pathways of cotton initiation, the DEGs in this study were compared against the Plant TFDB [40]. A total of 106 transcription factors were identified. Most belonged to MYB, WRKY, ERF HD-ZIP, and MYB-related families, with a small number of transcripts matching bHLH, bZIP, C3H, and C2H2 families. Transcription factors play important regulatory roles in various stages of cotton fiber development, beginning with initiation and continuing through secondary cell wall synthesis and maturity. In recent years, many transcription factors related to cotton fiber development have been reported, including MYB, HD-ZIP, and bHLH families. A model involving a MYB2/25-DEL61/65-TTG1/2 (MYB-bHLH-WD40) protein complex has been proposed to explain cotton fuzz initiation, similar to Arabidopsis root hair and trichome formation [41]. Given the importance of fiber initiation for fiber yield, some transcription factors have been considered in previous studies. *GhMYB25*, a good candidate gene for an association study of cotton fiber initiation, was highly expressed in 0-DPA ovules in our study. Moreover, the promoter of *GhMYB25* activated the GUS reporter in ovule epidermal cells of 0-DPA and 10-DPA fibers in a previous study [42]. Silencing of *GhMYB25* resulted in fewer fiber initials (10–20%) and inhibited fiber cell expansion as well as elongation. In contrast, over-expression of *GhMYB25* led to more fiber initials (15–35%) [42].

Nonsense mutations introduce premature stop codons into genes and can lead to an incomplete, potentially harmful, and usually nonfunctional protein product. Consequently, nonsense mutations are associated with disease susceptibility in humans and seem to be disadvantageous [43]. Some human diseases, such as thalassemia and Duchenne muscular dystrophy, result from nonsense mutations [44]. SNPs involving nonsense mutations have rarely been reported in plants. In this study, a nonsense SNP was identified in an auxin-responsive GH3 family protein (CotAD_19609). Light plays an important role in the regulation of plant developmental processes through complex signaling networks. In Arabidopsis, an auxin-responsive GH3 gene can mediate phytochrome B-regulated light signals during hypocotyl growth [45]. In *Triticum aestivum*, another early auxin-responsive gene is induced by light, calcium, and epibrassinolide [46]. Indole-3-acetic acid (IAA), the most important phytohormone, is required for fiber initiation from unfertilized ovules in culture [47]. Application of IAA *in vitro* can promote fiber initiation and increase total fiber units. In a previous investigation, the IAA biosynthetic gene *iaaM* driven by the petunia floral binding protein 7 gene promoter caused IAA to accumulate in cotton ovule epidermal cells from –2 to 10 DPA and also increased the number of lint fibers [48].

## Conclusions

Using the Illumina sequencing platform, we performed a deep transcriptome sequencing of cotton ovules during and prior to flowering and identified a number of potential SNP markers. The identified SNPs should serve as an important genetic resource for use in gene-based linkage mapping studies in cotton. In particular, the identification of SNPs from DEGs between a wild-type *G*. *hirsutum* cultivar and its *fl* mutant in this study should facilitate cloning of fiber-initiation-related genes and related functional genomic studies in cotton.

## Supporting Information

S1 Table Primers used for SNP validation. (XLSX)

S2 Table Single nucleotide polymorphisms (sheet 1) and insertions/deletions (sheet 2) identified in this study. (XLSX)

S3 Table Differentially expressed genes uncovered in this study. (XLSX)
